# Facial Erysipelas Following Pressurized Nasal Irrigation in Chronic Sinusitis: A Case Report

**DOI:** 10.7759/cureus.112359

**Published:** 2026-07-09

**Authors:** Mohammed Abrahim, Nour Abdelaziz

**Affiliations:** 1 Division of Emergency Medicine, Department of Family Medicine, McMaster University, Hamilton, CAN; 2 Emergency Department, Niagara Health System, Welland, CAN; 3 Medicine, University of Nicosia, Egkomi, CYP

**Keywords:** device-related infection, erysipelas, nasal lavage, over-the-counter products, pressurized nasal irrigation devices, sinusitis complication, skin adverse reaction

## Abstract

Facial erysipelas is an uncommon but potentially serious manifestation of rhinosinusitis, as involvement of the facial "danger triangle" carries the risk of orbital and intracranial complications. Saline nasal irrigation devices are widely recommended as nonprescription, over-the-counter adjunctive therapy for the symptomatic management of sinonasal disease and are generally considered safe. We report the case of a 60-year-old man who developed rapidly progressive centrofacial erysipelas after one week of using a commercially available pressurized seawater nasal irrigation device for an acute exacerbation of chronic rhinosinusitis. To our knowledge, this is the first reported temporal association between the use of a pressurized nasal irrigation device (PNID) and facial erysipelas. Although causality cannot be inferred from a single case, several biologically plausible mechanisms warrant consideration. This report highlights facial erysipelas as a rare cutaneous manifestation of complicated rhinosinusitis and underscores the importance of obtaining a detailed history of PNID use. Further studies are needed to clarify whether this observed association is causal or incidental and to better define the safety of PNIDs in patients with suspected bacterial rhinosinusitis.

## Introduction

Rhinosinusitis is a common disorder of the paranasal sinuses, most commonly associated with viral or bacterial pathogens [1]. Although most cases remain confined to the sinonasal cavity, the close anatomical relationship between the paranasal sinuses and adjacent facial, orbital, and intracranial structures allows infection to extend beyond the sinuses, resulting in potentially serious complications [2]. Involvement of the central facial “danger triangle” is of particular concern because of the potential for intracranial extension through valveless venous communications with the cavernous sinus [3].

Facial erysipelas is an acute infection involving the superficial dermis and lymphatic system, classically characterized by rapidly progressive erythema, warmth, edema, and sharply demarcated borders [4]. Notably, differentiating erysipelas from cellulitis remains challenging in clinical practice, partly due to inconsistent definitions and overlapping terminology in the literature. Traditionally, erysipelas is described as an infection of the upper dermis and superficial lymphatics, whereas cellulitis involves the deeper dermis and subcutaneous tissue. However, confirming this anatomical distinction at the bedside is often impractical, and diagnosis relies primarily on clinical features rather than definitive tissue localization. Although erysipelas is classically associated with more sharply demarcated inflammation, significant clinical overlap exists between these entities. Further complicating this distinction, some authors use erysipelas specifically to describe facial cellulitis, while others, particularly in European literature, consider erysipelas and cellulitis to be synonymous [4].

Treatment of complicated rhinosinusitis, particularly when associated with facial soft tissue infection, requires antimicrobial therapy targeting common pathogens, with consideration of methicillin-resistant Staphylococcus aureus (MRSA), gram-positive organisms, gram-negative organisms, and anaerobes when clinically indicated. Empiric intravenous regimens may include vancomycin for MRSA coverage, combined with agents such as ceftriaxone, cefotaxime, ampicillin-sulbactam, or piperacillin-tazobactam, with the addition of metronidazole when enhanced anaerobic coverage is required. Alternative MRSA-active agents include daptomycin, linezolid, and telavancin, although clinical experience with these agents is more limited in orbital or intracranial infections. In patients with significant beta-lactam allergy, vancomycin combined with fluoroquinolones such as ciprofloxacin or levofloxacin may be considered. Following clinical improvement, transition to targeted or empiric oral therapy may be appropriate, with antibiotic selection guided by microbiological data, clinical response, and local resistance patterns [5].

Nasal saline irrigation is widely recommended as adjunctive therapy for acute-on-chronic rhinosinusitis and is generally considered safe [6]. Pressurized nasal irrigation devices (PNIDs) provide high-flow saline delivery throughout the nasal cavity; however, their potential association with infectious complications remains poorly described [7]. We report a case of facial erysipelas occurring in a patient with severe acute-on-chronic rhinosinusitis shortly after the initiation of a pressurized seawater nasal irrigation device.

## Case presentation

A 60-year-old man presented to the emergency department with a two-day history of rapidly progressive facial erythema, pain, headache, and fever. His medical history was significant for chronic rhinosinusitis. One week before presentation, he was evaluated by his primary care physician for worsening sinonasal symptoms and was advised to use a commercially available pressurized seawater nasal irrigation spray three times daily for one week. Several days after initiating nasal irrigation therapy, he developed progressive erythema involving the central face. He denied regular medication use and had no known drug allergies.

On presentation, the patient appeared nontoxic and was afebrile. His heart rate was 104 beats/min, while all other vital signs were within normal limits. Physical examination demonstrated bilateral, symmetrical, sharply demarcated erythema, warmth, and edema involving the malar regions and nasal bridge, with extension toward the periorbital areas (Figure 1). There was no fluctuance, purulent drainage, crepitus, or skin necrosis. The remainder of the physical examination was otherwise unremarkable.

**Figure 1:**
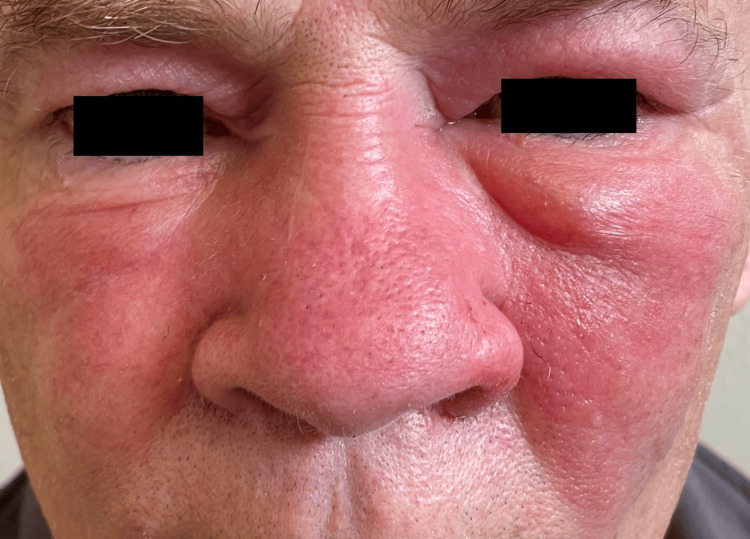
Physical examination findings The image shows bilateral erythema and swelling of the malar regions and nasal bridge extending toward the periorbital region, consistent with centrofacial erysipelas involving the facial danger triangle

Laboratory investigations revealed leukocytosis (13 × 10⁹/L) with neutrophilia. Blood cultures were obtained. Given the patient's headache and the involvement of the central facial “danger triangle,” CT of the head with intravenous (IV) contrast was performed to exclude deep soft-tissue infection, orbital extension, and cavernous sinus thrombosis. CT imaging demonstrated diffuse facial soft-tissue swelling and severe bilateral inflammatory changes of the paranasal sinuses, including near-complete opacification of the right maxillary sinus, consistent with acute-on-chronic rhinosinusitis (Figure 2). No evidence of orbital involvement, intracranial extension, abscess formation, or cavernous sinus thrombosis was identified.

**Figure 2:**
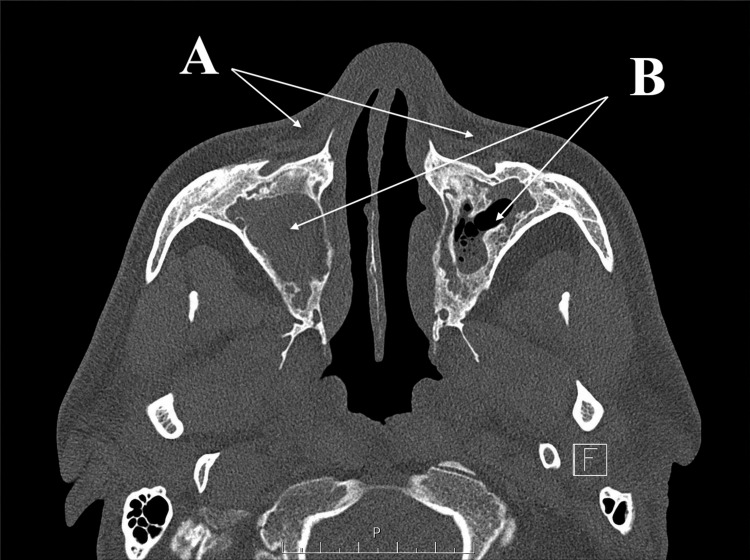
Axial contrast-enhanced CT image of the head The image demonstrates diffuse bilateral facial soft-tissue swelling involving the central face and malar regions (A), with severe bilateral maxillary sinus inflammatory changes (B). There is near-complete opacification of the right maxillary sinus and mucosal thickening of the left maxillary sinus, consistent with acute-on-chronic rhinosinusitis CT: computed tomography

Empiric intravenous piperacillin-tazobactam was initiated for broad-spectrum antimicrobial coverage and subsequently de-escalated to ceftriaxone after blood cultures demonstrated no growth. Use of PNID was discontinued. The patient was managed through an outpatient parenteral antibiotic therapy program, returning daily for antibiotic administration and clinical reassessment. Facial erythema, pain, and swelling progressively improved, with substantial clinical resolution. He was subsequently transitioned to oral amoxicillin-clavulanate to complete therapy and was referred for otolaryngology follow-up.

## Discussion

Facial erysipelas is an acute superficial bacterial skin infection characterized by rapidly progressive, sharply demarcated erythema, edema, warmth, and tenderness. It is most commonly caused by Streptococcus pyogenes and typically develops following bacterial entry through disruption of the skin or mucosal barrier [4]. Although lower extremity involvement is more common, facial erysipelas remains clinically important because infections involving the central face may rarely progress through valveless facial venous pathways, creating a potential risk for orbital and intracranial complications [3].

This report describes an unusual temporal association between the use of a PNID and the subsequent development of facial erysipelas in a patient with acute-on-chronic rhinosinusitis. Nasal saline irrigation is widely recommended as an adjunctive therapy for rhinosinusitis because it improves mucociliary clearance, facilitates removal of secretions, and may reduce local inflammatory mediators [1,6]. It is generally considered safe, with reported adverse effects usually limited to minor symptoms such as nasal irritation, discomfort, and epistaxis [6,7]. However, nasal irrigation systems differ substantially in their delivery mechanisms, pressure generation, and flow dynamics. While low- and moderate-pressure irrigation methods have established safety profiles, the potential effects of high-flow pressurized systems during episodes of active bacterial rhinosinusitis remain poorly characterized [7].

The relationship between PNID use and facial erysipelas in this case cannot establish causality, and several interpretations should be considered. The most conservative explanation is that erysipelas represented a complication of acute bacterial rhinosinusitis, with PNID exposure being an incidental association. Alternatively, pressurized irrigation could theoretically contribute to infectious spread in susceptible individuals by generating pressure-driven movement of contaminated secretions, producing microtrauma to an already inflamed sinonasal mucosa, or facilitating bacterial translocation across compromised mucosal barriers. The close anatomical relationship between the nasal cavity, paranasal sinuses, periorbital tissues, and facial venous drainage provides a biologically plausible route for contiguous inflammatory or infectious extension [2]. Nevertheless, these proposed mechanisms remain speculative and require further investigation.

The clinical presentation in this case was notable for bilateral and relatively symmetrical involvement of the malar regions and nasal bridge. Although erysipelas classically presents as a unilateral infection, facial involvement may extend across midline structures because of regional lymphatic and soft-tissue continuity. The differential diagnosis includes cellulitis, allergic or irritant contact dermatitis, angioedema, rosacea exacerbation, autoimmune malar eruption, and other inflammatory dermatoses. In this patient, acute pain, warmth, progressive erythema, leukocytosis with neutrophilia, and radiographic evidence of significant acute-on-chronic rhinosinusitis supported an infectious etiology.

This report also highlights the importance of careful evaluation of infections involving the central facial “danger triangle.” Although uncommon, complications such as orbital cellulitis, abscess formation, septic cavernous sinus thrombosis, and intracranial extension carry significant morbidity [3]. In patients presenting with facial erysipelas accompanied by concerning features such as severe headache, periorbital involvement, neurological symptoms, or evidence of severe sinonasal disease, cross-sectional imaging should be considered. In the present case, contrast-enhanced CT excluded orbital and intracranial complications and confirmed extensive sinonasal inflammation, allowing appropriate antimicrobial management.

The findings of this case should not discourage the use of nasal saline irrigation, which remains an evidence-supported intervention for selected patients with rhinosinusitis. Rather, it emphasizes the importance of appropriate patient education regarding device selection, hygiene practices, irrigation technique, and avoidance of excessive pressure, particularly during episodes of severe acute sinonasal inflammation. Clinicians should consider obtaining a detailed history of nasal irrigation practices, including device type, pressure characteristics, frequency of use, and temporal relationship to symptom development.

To our knowledge, this is the first reported case describing facial erysipelas occurring shortly after PNID use in a patient with acute-on-chronic rhinosinusitis. Additional case reporting and prospective studies are required to determine whether pressurized nasal irrigation represents a potential contributing factor, a precipitating event in susceptible individuals, or simply a coincidental exposure during the natural progression of bacterial rhinosinusitis.

## Conclusions

This report describes a previously unreported temporal association between the use of a pressurized seawater nasal irrigation device and the development of facial erysipelas in a patient with acute-on-chronic rhinosinusitis. Although causality cannot be inferred, the observation highlights facial erysipelas as a rare cutaneous manifestation of complicated rhinosinusitis and underscores the importance of maintaining a high index of suspicion for underlying sinonasal disease in patients presenting with infection of the facial danger triangle. Careful assessment of nasal irrigation practices and early cross-sectional imaging may facilitate timely diagnosis and exclusion of potentially life-threatening orbital or intracranial complications. Further clinical and mechanistic studies are needed to clarify whether this association is causal or coincidental and to better define the safety of PNIDs in the setting of bacterial rhinosinusitis.
